# Dynamic network curvature analysis of gene expression reveals novel potential therapeutic targets in sarcoma

**DOI:** 10.1038/s41598-023-49930-4

**Published:** 2024-01-04

**Authors:** Rena Elkin, Jung Hun Oh, Filemon Dela Cruz, Larry Norton, Joseph O. Deasy, Andrew L. Kung, Allen R. Tannenbaum

**Affiliations:** 1 Department of Medical Physics, Memorial Sloan Kettering Cancer Center, New York, 10065 USA; 2 Department of Pediatrics, Memorial Sloan Kettering Cancer Center, New York, 10065 USA; 3 Department of Medicine, Memorial Sloan Kettering Cancer Center, New York, 10065 USA; 4 Departments of Computer Science and Applied Mathematics and Statistics, Stony Brook University, Stony Brook, 11794 USA

**Keywords:** Cancer, Computational science

## Abstract

Network properties account for the complex relationship between genes, making it easier to identify complex patterns in their interactions. In this work, we leveraged these network properties for dual purposes. First, we clustered pediatric sarcoma tumors using network information flow as a similarity metric, computed by the Wasserstein distance. We demonstrate that this approach yields the best concordance with histological subtypes, validated against three state-of-the-art methods. Second, to identify molecular targets that would be missed by more conventional methods of analysis, we applied a novel unsupervised method to cluster gene interactomes represented as networks in pediatric sarcoma. RNA-Seq data were mapped to protein-level interactomes to construct weighted networks that were then subjected to a non-Euclidean, multi-scale geometric approach centered on a discrete notion of curvature. This provides a measure of the functional association among genes in the context of their connectivity. In confirmation of the validity of this method, hierarchical clustering revealed the characteristic *EWSR1*-*FLI1* fusion in Ewing sarcoma. Furthermore, assessing the effects of in silico edge perturbations and simulated gene knockouts as quantified by changes in curvature, we found non-trivial gene associations not previously identified.

## Introduction

Genes function in networks to control all aspects of a cell’s biology, including the morphologic and behavioral aberrations of cancer cells^1^. Hence, to identify meaningful therapeutic targets, biomarkers of prognosis, or sensitivity to drugs, it is critical to gain an understanding not just of gene function but also of the networks in which they are active. Regulatory networks are commonly represented as weighted graphs in which each gene is represented as a node (vertex), with edges between nodes representing direct interactions at the protein level. The strength of the interactions is estimated by the weights of the corresponding edges. In addition to direct connections, indirect cooperation occurs, and therefore it is essential for a useful method to identify these as well. However, identifying relevant subnetworks in complex biological networks remains challenging, with existing methods possibly missing potential therapeutic targets. To overcome this barrier, we have developed, and in this paper apply, a method that utilizes a geometric approach, namely curvature, founded on concepts from optimal mass transport (OMT) theory^2,3^, in combination with analysis of network dynamics.

Representing a weighted network as a Markov chain, one can consider certain graph theoretical properties such as random walks. Of particular interest is the notion of Ricci curvature between two nodes on a graph. In a continuous setting, curvature is a measure of how the local geometry deviates from Euclidean space. Intuitively, curvature is characterized by the degree to which geodesics (local paths of minimal length), obtained via parallel transport, will tend to converge or diverge in the space^4^. A standard example of a positively curved space is a sphere whose geodesics trace out the great circles (Fig. S1). In the context of networks, curvature reflects the connectivity and interdependence among nodes. Several notions of discrete Ricci curvature applicable to graphs have been proposed^5,6^, each with its according advantages and disadvantages. We chose to employ Ollivier’s formulation^7^, which we simply refer to as *Ollivier-Ricci curvature*, due to several considerations, which we now outline.

In the present study, we employed a dynamical model of curvature^8^, which is based on starting with delta functions at each node, then progressively smoothing via the heat flow defined by the graph Laplacian, computing the resulting Wasserstein distances, and finally the Ollivier-Ricci curvature. (This process is described below.) This allows us to geometrically study the network at various scales. Out of all the versions of discrete curvature, the Ollivier-Ricci approach is the most natural for the type of dynamical model we utilize in this paper. There are a number of other useful properties of the Ollivier-Ricci curvature. Complete sets of references may be found in^9,10^. These include the connection of Ollivier-Ricci curvature to the number of invariant triangles and thus network feedback stability, connections to stochastic systems and the rate function for convergence to a stationary state, convergence to equilibrium and mixing times for Markov chains, and the positive correlation of curvature to changes in entropy and system functional robustness. All of these heavily rely on the optimal mass transport underpinnings of the Oliver-Ricci model.

The recently developed dynamic formulation of Ollivier-Ricci curvature^8^ seems to provide an excellent way to explore the multi-scale structure of genomic networks and identify key subgraphs as well as the bridges connecting them. In the dynamic setting, curvature is measured as a function of time while information is diffused throughout the network. ”Time” in this context is a purely numerical construct used to connote the gradations of the network organization and is used interchangeably with *scale*. The motivation is that networks exhibit varying levels of organization at different scales. Thus, persisting communities (with many connections among genes) and emerging bridges (with few connections) may identify mechanisms of drug resistance and actionable targets for intervention. This dynamic notion of curvature is applicable to networks in general and is particularly attractive for gene regulatory networks that typically have strong hub nodes and low modularity, which is challenging to overcome with standard community detection approaches. We demonstrate its utility with particular application in pediatric sarcoma (PS).

PSs are a diverse group of childhood cancers that are typically diagnosed based on immunohistologic features and clinical history^11^. When the clinical and histologic workup do not unequivocally determine a diagnosis, further time-intensive molecular characterization is needed to ascertain the correct classification^12^. The delay in a definitive diagnosis hinders time-sensitive decisions toward treatment planning and management. Therefore, there is a significant need to develop novel methodologies to accelerate the timeline for identifying PS subtypes. Moreover, although the genetic drivers for some PS subtypes have been described^13^, oncogenic driver mutations, like the canonical *EWSR1/FLI1* fusion gene characteristic of Ewing sarcoma (EWS), have not been amenable to direct targeting and are therefore undruggable^14–20^. Thus, understanding the pathways required to maintain the cancer system is also pivotal to the identification of existing drugs that can indirectly target the drivers of these tumors.

The goal of this study was two-fold: to distinguish PS subtypes from tumor tissue RNA-Seq gene expression profiles and identify actionable candidate targets for therapeutic intervention. To this end, the focus of the work described in this paper is to design a classifier for identifying PS subtypes and to develop a framework for investigating the functional relationships between genes or their products. Machine learning techniques such as agglomerative hierarchical clustering methods^21,22^ and random forest models^22,23^ have had success in classifying sarcoma tumors and statistical analyses of differential patterns in gene expression (or methylation) between subtypes have been particularly useful for identifying novel biomarkers. In this work, we exploit functional network properties that consider the topology (connectivity) of biological networks in conjunction with gene expression to address each objective. More specifically, we employ curvature^2,3^, which has not been fully explored in the context of weighted cancer networks. Curvature defined on a graph in this manner is related to the feedback connectivity, i.e., the number of invariant triangles^24^. Informally, curvature provides insight on the shape of the interactome landscape, analogous to a surface, by quantifying how easy (or difficult) it is to transport information between genes over the network. By accounting for the network topology and gene-prescribed weights, such a geometric, functional network representation allows for novel insight that is not apparent from genomic data alone.

Moreover, Ollivier’s notion of Ricci curvature is relevant to studying network functional stability because an increase in Ollivier-Ricci curvature (resulting from an external impact exhibited by a change in interaction (strength) between network components) is positively correlated to an increase in system robustness^25,26^, meaning that an increase in curvature indicates an increase in functional connectivity on the network associated with gene-cooperation. The connection between curvature and network robustness/fragility is linked by entropy^25^. However, unlike entropy which is a nodal attribute and thereby exhibits a loss of information by construction due to a weighted contraction of edge dependencies, Ricci curvature is an edge attribute that preserves such geometric quantities. The significance of this theoretical result has been demonstrated on real networks supporting the use of curvature as an indicator of network robustness^9,10^. Thus, curvature concurrently computed with *in silico* experiments simulating gene knockout or pathway interference is performed to assess the network response to targeting the key contributors to gene signaling dysregulation in the cancer network identified by the multi-scale dynamical analysis, with particular attention in this work given to EWS.

## Methods

### Data

RNA-Seq data were generated from tumor tissues in PS patients who were treated at our institute. RNA-Seq data were preprocessed using regularized log (rlog) normalization prior to analysis. This study was approved by Internal Review Board at Memorial Sloan Kettering Cancer Center. The patients provided their written informed consent to participate in this study and all methods were performed in accordance with the relevant guidelines and regulations. In total, the cohort consisted of 102 samples from 21 different subtypes that were predominantly sequenced from metastatic or relapsed tumors. In this work, we considered the 70 samples from the four largest subtypes: osteosarcoma (OST; $n=29$), desmoplastic small round cell tumor (DSRCT; $n=20$), EWS ($n=12$) and embryonal rhabdomyosarcoma (embryonal RMS; $n=9$) and concentrated on the EWS cohort for functional analysis. The criterion for gene inclusion was a minimum of 10 samples with 10 read counts in RNA-Seq data.

### Graph topology

The network topology was derived from the Human Protein Reference Database (HPRD)^27,28^. The graph was then constructed by restricting the set of genes in the given dataset to the HPRD and extracting the largest connected component network, resulting in a simple graph with 8,127 nodes, 32,750 edges, and an average degree of 8.1 after removing multi-edges and self-edges. ${\mathscr {G}}_C$ is used to denote the graph used for tumor clustering while ${\mathscr {G}}$ is used when referring to an arbitrary graph, which is assumed to be simple, undirected and connected. As the nodes of the graph refer to genes, the terms *gene* and *node* are used interchangeably.

### Unsupervised sample clustering

We follow the construction of forming a Markov chain based on RNA-Seq gene expression data^29^. The Ollivier-Ricci curvature is defined on such a Markov chain as we will describe below.

Gene expression data $x\in {\mathbb {R}}^n$ for each sample is mapped to the graph ${\mathscr {G}}=({\mathscr {V}},{\mathscr {E}})$ where ${\mathscr {V}}$ denotes the set of *n* nodes and ${\mathscr {E}}$ denotes the set of edges by assigning node weights $w_i = x_i$ for all nodes $i \in {\mathscr {V}}$. Treating the weighted graph as a Markov chain, the probability of going from node *i* to node *j* on a random walk is expressed as

1 $$\begin{aligned} P_{ij} = {\left\{ \begin{array}{ll} \frac{w_j}{\sum \limits _{k\in {\mathscr {N}}_i}w_k}, \; &{} \textrm{if} \; j\in {\mathscr {N}}_i\\ 0, \; &{} \textrm{otherwise}, \end{array}\right. } \end{aligned}$$

where ${\mathscr {N}}_i$ denotes the neighborhood of node *i*: ${\mathscr {N}}_i = \{j\in {\mathscr {V}} | (i,j)\in {\mathscr {E}}\}$. The random walk on ${\mathscr {G}}$ with a transition probability matrix *P* corresponds to an irreducible Markov chain since ${\mathscr {G}}$ is connected. This along with the Perron-Frobenius theorem for nonnegative matrices guarantees the existence of a unique *stationary distribution* $\pi$, which is the probability distribution defined on ${\mathscr {V}}$ that satisfies

2 $$\begin{aligned} \pi P = \pi . \end{aligned}$$

The stationary distribution may be efficiently computed from its closed form

3 $$\begin{aligned} \pi _i = \frac{1}{K}w_i\sum \limits _{j\in {\mathscr {N}}_i}w_j, \end{aligned}$$

where *K* is a normalization factor.

The stationary distribution is the limiting behavior of a random walk on ${\mathscr {G}}$ and the value $\pi _i$ of its *i*-th component is related to the relative amount of time a random walker spends at the corresponding node *i*. We expect that the stationary distribution encodes subtype-specific relative node importance and therefore expect that stationary distributions associated with transition matrices, constructed from gene expression data of samples with the same subtype, would be more similar than those associated with different subtypes. This motivates the use of the *Wasserstein distance* $W_1$, the metric associated with OMT which gives a rigorous notion of the “shortest distance” between probability distributions, to compute the distance between stationary distributions as a measure of similarity between the corresponding samples. The Wasserstein distance between two discrete probability distributions $\mu$ and $\nu$ on ${\mathbb {R}}^n$ is formally expressed as

4 $$\begin{aligned} W_1(\mu ,\nu ) = \inf _{\gamma \in \Gamma (\mu ,\nu )}\sum _{i,j}\gamma _{ij}d_{ij}, \end{aligned}$$

where $\Gamma (\mu ,\nu )$ denotes the set of joint probabilities on ${\mathbb {R}}^n\times {\mathbb {R}}^n$ with marginals $\mu$ and $\nu$ and $d_{ij}$ is the prescribed distance between the corresponding genes *i* and *j*. For details on the Wasserstein distance, more general formulations and its connection to OMT, see^2,3,30^.

The unsupervised Wasserstein distance-based clustering of the samples proceeds in the following manner: invariant distributions $\pi ^{(s)}$ are computed for each sample *s*, $s=1,\ldots ,S$ where *S* is the number of samples. The sample-pairwise Wasserstein distance matrix ${\textbf{W}}\in {\mathbb {R}}^{S\times S}$ is then computed where ${\textbf{W}}_{qr} = W_1(\pi ^{(q)},\pi ^{(r)})$ is the Wasserstein distance between the stationary distributions associated with samples *q* and *r* using the hop distance as the graph metric $d_{ij}$. Hierarchical clustering is then performed using ${\textbf{W}}$ as the distance matrix.

### Geometric network analysis

#### Graph construction

The graph for functional analysis ${\mathscr {G}}_F$ was constructed by extracting the largest connected component from ${\mathscr {G}}_C$ restricted to the set of genes provided by the OncoKB database^31^, resulting in a simple graph with 675 nodes, 2,667 edges and an average degree of 7.9. Note that the analysis performed in this work may also be applied to the full ${\mathscr {G}}_C$ as well. The constricted network of established oncogenes and tumor suppressor genes was opted for to reduce the computational burden.

For each PS subtype, the strength of interaction on an edge $(i,j)\in {\mathscr {E}}$, denoted ${\tilde{w}}_{ij}$, was computed as

5 $$\begin{aligned} {\tilde{w}}_{ij} = |c_{ij}|, \end{aligned}$$

where $c_{ij}$ is the Pearson correlation between the corresponding genes *i* and *j*. Pearson correlation is known to be sensitive to outliers so a de-sensitized correlation was computed where samples that drastically affected the correlation value were removed. Mapping the interaction strengths ${\tilde{w}}$ to edge weights, as described in Equation 8, on the fixed ${\mathscr {G}}_F$ topology yielded the subtype-specific weighted graph.

#### Graph distance

Unless specified otherwise, the graph distance *d* is hereon assumed to be the *weighted hop distance* $d^w$ (i.e., $d\equiv d^w$). More specifically, denote by $p^{ij}$ a path between nodes *i* and $j \in {\mathscr {V}}$ by the set of $m+1$ nodes connecting them, i.e., $p^{ij}:= i=v_0 \sim v_1 \sim \cdot \cdot \cdot \sim v_m=j$, where consecutive nodes $v_k,v_{k+1}\in p^{ij}$ ($k=0,1,\ldots ,m-1$) correspond to an edge $e_k = (v_k,v_{k+1})\in {\mathscr {E}}$ and each node only appears once. Denoting the set of all possible paths between *i* and *j* by ${\mathscr {P}} = \{p_0^{ij}, p_1^{ij},\ldots , p_r^{ij}\}$ (this set is finite since the graph is finite), let $\{w_0^s,w_1^s,\ldots w_{m-1}^s\}$ be the set of edge weights associated with path $p_s^{ij}\in {\mathscr {P}}$ where $w_k^s \equiv w^s_{k(k+1)}$ is the weight for edge $e^s_k = (v_k,v_{k+1})$. The corresponding length of the path is then expressed as

6 $$\begin{aligned} \ell (p_s^{ij}) = \sum \limits _{k=0}^{m-1}\frac{1}{\sqrt{w_k^s}}. \end{aligned}$$

The weighted hop distance $d^w_{ij}$ between nodes *i* and $j \in {\mathscr {V}}$ is the minimal accumulated edge weight among all paths connecting *i* and *j* formally defined as

7 $$\begin{aligned} d^w_{ij}:= \min \limits _{0\le s\le r} \ell (p_s^{ij}). \end{aligned}$$

The graph under consideration is assumed to be simple, connected and undirected so at least one path is guaranteed to exist between any two nodes $i,j\in {\mathscr {V}}$. For each edge $(u,v)\in {\mathscr {E}}$, the edge weight $w_{uv}$ is taken to be

8 $$\begin{aligned} w_{uv} = \frac{1}{\sqrt{{\tilde{w}}_{uv}}}, \end{aligned}$$

where ${\tilde{w}}_{uv}$ was previously prescribed in Equation (5).

#### Ollivier–Ricci graph curvature

Treating a graph as a metric measure space equipped with a graph metric *d* and probability measures $\mu _k$ at each node $k\in {\mathscr {V}}$, Ollivier’s^7,26^ coarse definition of curvature between any two nodes $i,j\in {\mathscr {V}}$ is expressed as

9 $$\begin{aligned} \kappa (i,j) = 1 - \frac{W_1(\mu _i,\mu _j)}{d_{ij}}. \end{aligned}$$

One possibility is to take the distribution $\mu _k$ to be the probability of a 1-step random walk starting at node *k* given by $P_k$, *i*.*e*.,  the *k*-th row of the transition matrix *P* in Equation 1. Alternatively, distributions based on lazy walks or edge weights may be used. As mentioned previously, Ricci curvature on a Riemannian manifold can be assessed by the local tendency of geodesics to converge (positive curvature) or diverge (negative curvature)^4^. Put another way, curvature may be characterized by the ratio of the distance between geodesic balls to the distance between their centers: positive (respectively, negative) curvature is characterized by the distance between geodesic balls (on average) being closer (respectively, farther) than their centers. The ratio is balanced, meaning the distance between geodesic balls is the same as the distance between their centers, in *flat* space, e.g., Euclidean space. In Equation 9, Ollivier’s definition replaces geodesic balls centered at a point with distributions supported on a node’s neighborhood and the Wasserstein distance is kindred to the distance between geodesic balls. Thus, analogous to Ricci curvature, Ollivier-Ricci curvature is characterized by the ratio of the distance between neighborhoods to the graph distance between the nodes the distributions are centered on.

#### Dynamic curvature

In this paper, we employ a multi-scale extension of the Ollivier-Ricci curvature on weighted graphs to identify robust and fragile components of the genomic network that are obscured by the complexity (non-linear, non-Euclidean) of the network representation^8^. The multi-scale functional organization is captured by replacing the random walk $\mu _i$ with a network diffusion process $\eta _i(\tau )$ as a function of scale $\tau \in [0, T]$ seeded at individual nodes *i*, expressed as

10 $$\begin{aligned} \eta _i(\tau ):= \delta _i\exp ^{-L\tau }, \end{aligned}$$

where $\delta _i$ is the Dirac measure at node *i* such that $\delta _i(j) =1$ for $i=j$ and 0 otherwise, and $L=I-K^{-1}A$ is the (random-walk) normalized graph Laplacian. To construct *L*, *I* is the $n\times n$ identity matrix where *n* is the number of nodes in the network, *K* is the diagonal degree matrix where $K_{ii} = \sum _j A_{ij}$ and *A* is the weighted adjacency matrix. In this work, *A* is defined as

11 $$\begin{aligned} A_{ij} = {\left\{ \begin{array}{ll} d_{max} - d_{ij}, \, &{} \textrm{if}\, (i,j)\in {\mathscr {E}}\\ 0, \, &{} \textrm{otherwise}, \end{array}\right. } \end{aligned}$$

where $d_{max} = \max _{ij}d_{ij}$ is the largest distance. Accordingly, Gosztolai and Arnaudon^8^ define a *dynamic* version of Ollivier-Ricci curvature as

12 $$\begin{aligned} \kappa _{ij}(\tau )= 1- \frac{W_1(\eta _i(\tau ), \eta _j(\tau ))}{d_{ij}}. \end{aligned}$$

**Figure 1:**
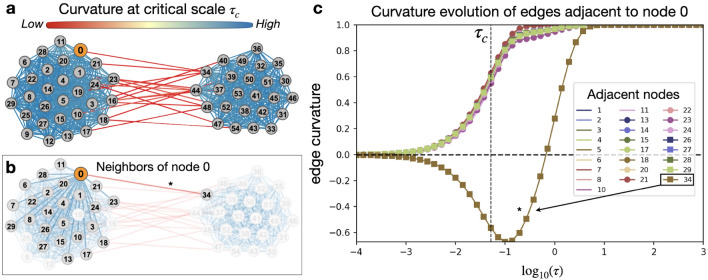
Utility of the dynamic curvature framework illustrated on an idealized stochastic block model network with two communities. (**a**) Bridges between clusters characteristically have negative curvature (red) while edges within clusters are positive (blue). (**b**) Multi-scale functional organization exhibited for node 0 is encoded by (**c**) the curvature evolution of incident edges, seen by the largest gap obtained in the evolution of the bridge edge (0,34), denoted with an asterisk, that connects the two communities.

Notice that initially, the dynamic curvature is 0 at $\tau =0$ ($\kappa _{ij}(0)=0)$ when no information has been shared and the nodes are independent. Then when the measures diffuse to steady state $\pi$, and the diffusion processes have completely mixed, one gets that $\kappa _{ij}(\tau )=1$. The key idea, as the authors argue, is that the characteristic scales should be related to the overlap of pairs of diffused measures (a.k.a. the mixing rate) over the network. This is used as a measure of information propagation on the various subnetworks. Indeed, they derive an upper bound on the mixing time of the diffusion pair. Thus, information shared to ”communal” neighbors is reflected by clusters with positive curvature at early times, whereas negative curvature is characteristic of inter-community connections (bridges) with restricted information exchange (Fig. 1).

#### Critical curvature filter

In addition to the multi-scale representation, there is also a hierarchical aspect within a fixed scale, as curvature measures the strength of the functional connections. The first scale that the dynamic curvature of an edge reaches a critical value, here set to 0.75, is called the *critical scale* $t_c$, i.e., $\kappa _{ij}(t_c) = 0.75$. The critical scale based on this critical value is not arbitrary; it is related to the scale at which information has sufficiently diffused throughout communities but has not crossed bridge (bottleneck) edges and is therefore an ideal scale to capture functional subnetworks^8^. Bridges may be identified as edges with negative curvature at the critical scale. Connected components that emerge by removing these bridges characterize communal affiliation amongst the nodes. Moreover, iteratively pruning edges by the critical curvature value in increasing order reveals a hierarchical structure of the functional association between nodes.

#### Multi-scale functional clustering

Incorporating information from multiple scales in the dynamic range lends additional information for characterizing the intricate fabric of the network and its key sub-structures. In order to utilize the multi-scale information, we define the *average critical curvature* ${\tilde{\kappa }}_{ij}$ of an edge (*i*, *j*) as the average curvature over the critical dynamic range, expressed as

13 $$\begin{aligned} {\tilde{\kappa }}_{ij}:= t_c^{-1}\sum _{\tau =0}^{t_c}\kappa (\tau ,i,j). \end{aligned}$$

In this manner, ${\tilde{\kappa }}$ provides an enhanced measure of the interaction between nodes. The edges of the network are then iteratively pruned by their ${\tilde{\kappa }}$ value, starting by removing all edges with negative ${\tilde{\kappa }}$ and then proceeding in a monotonically increasing order. We keep track of the number of iterations nodes *i* and *j* are found in the same connected component, denoted $R_{ij}$ for every two nodes $i,j\in {\mathscr {V}}$ in a *persistent component score matrix* $R\in {\mathbb {R}}^{n\times n}$, where *n* is the number of nodes. With the rationale that the longer two genes remain in the same connected component, the stronger their functional association, and the “closer” they are to each other. Accordingly, we construct a gene-pairwise distance matrix between nodes $D\in {\mathbb {R}}^{n\times n}$ where

14 $$\begin{aligned} D_{ij} = \max _{rs}R_{rs} - R_{ij}. \end{aligned}$$

Hierarchical clustering of the genes is then performed using *D* (Equation 14) as the distance matrix. This process of hierarchical clustering based on how often nodes are found in the same connected component while iteratively filtering out edges by the average critical curvature is illustrated in Fig. S2 and is referred to as hierarchical-acc.

#### Edge perturbation simulations

To assess the network response to targeting a particular edge, curvature is re-computed while dampening a specific edge-weight. Specifically, for a fixed edge $(i,j)\in {\mathscr {E}}$ with interaction strength ${\tilde{w}}_{ij}$ (Equation 5) and weighted hop distance computed from edge weights according to Equation 8, the baseline curvature between any two nodes is computed according to Equation 9. The nodal measure $\mu _r$ used for the baseline curvature computation is expressed as

15 $$\begin{aligned} \mu _r(s) = {\left\{ \begin{array}{ll} \frac{{\tilde{w}}_{rs}}{\sum \limits _{q\in {\mathscr {N}}_r}{\tilde{w}}_{rq}},\, &{} \textrm{if} \, s\in {\mathscr {N}}_r\\ 0, \, &{} \textrm{otherwise}. \end{array}\right. } \end{aligned}$$

The edge perturbation procedure then proceeds as follows. The interaction strength ${\tilde{w}}_{ij}$ is perturbed toward 0 to simulate a disruption in communication, or cooperation, between the nodes. Our interest is to see the trend in curvature due to the simulated reduction in cooperation. To reduce the computational time, we therefore choose a coarse discretization of the interval $[\epsilon ,{\tilde{w}}_{ij}]$ (where $\epsilon$ is a negligible amount, $1\times 10^{-6}$) into $N = 6$ uniformly spaced points: ${\hat{c}}_{ij}^{(\zeta )} = \epsilon + (\zeta -1)h, \; \zeta =1,\ldots ,6$, where *h* is the discretization step $h=({\tilde{w}}_{ij} - \epsilon )/(N-1)$. For each $\zeta =1,\ldots ,6$, the perturbed edge weight ${\hat{w}}_{ij}$ is computed as ${\hat{w}}_{ij}=1/\sqrt{{\hat{c}}_{ij}^{(\zeta )}}$ and the weighted hop distance is recomputed. Accordingly, we consider the ”perturbed” probability measures ${\hat{\mu }}_r$ attached to node $r\in {\mathscr {V}}$ expressed as

16 $$\begin{aligned} {\hat{\mu }}_{r}(s):= {\left\{ \begin{array}{ll} \frac{a_{rs}}{\sum \limits _{q\in {\mathscr {N}}_r}a_{rq}},\, &{} \textrm{if} \, s\in {\mathscr {N}}_r\\ 0,\, &{} \textrm{otherwise}, \end{array}\right. } \end{aligned}$$

where the edge attribute *a* is defined as

17 $$\begin{aligned} a_{rs} = {\left\{ \begin{array}{ll} {\hat{c}}_{ij}^{(\zeta )}, \, &{} \textrm{if}\, (r,s)=(i,j)\\ {\tilde{w}}_{rs}, \, &{} \textrm{otherwise}. \end{array}\right. } \end{aligned}$$

Finally, the *perturbed* Ollivier-Ricci curvature is then computed between any two nodes according to Equation 9.

#### Gene knockout simulations

To assess the network response to targeting a particular gene, curvature is re-computed after removing the corresponding node from the network. The baseline curvature between any two nodes is computed as previously described in “Edge perturbation simulations” section. The gene knockout procedure then proceeds as follows. For a fixed node $i\in {\mathscr {V}}$, node *i* is removed from the graph to create a subgraph ${\mathscr {G}}_S = ({\mathscr {V}}_S,{\mathscr {E}}_S)$, where ${\mathscr {V}}_S = {\mathscr {V}}\backslash \{i\}$ and ${\mathscr {E}}_S = {\mathscr {E}}\backslash \{(i,j) \,|\, j \in {\mathscr {N}}_i\}$. The *knocked-out* Ollivier-Ricci curvature is then computed between any two nodes in the subgraph ${\mathscr {G}}_S$ according to Equation (9), where the weighted hop distance and nodal measures are computed in the same manner as the baseline curvature.

## Results

### Sample clustering

**Figure 2:**
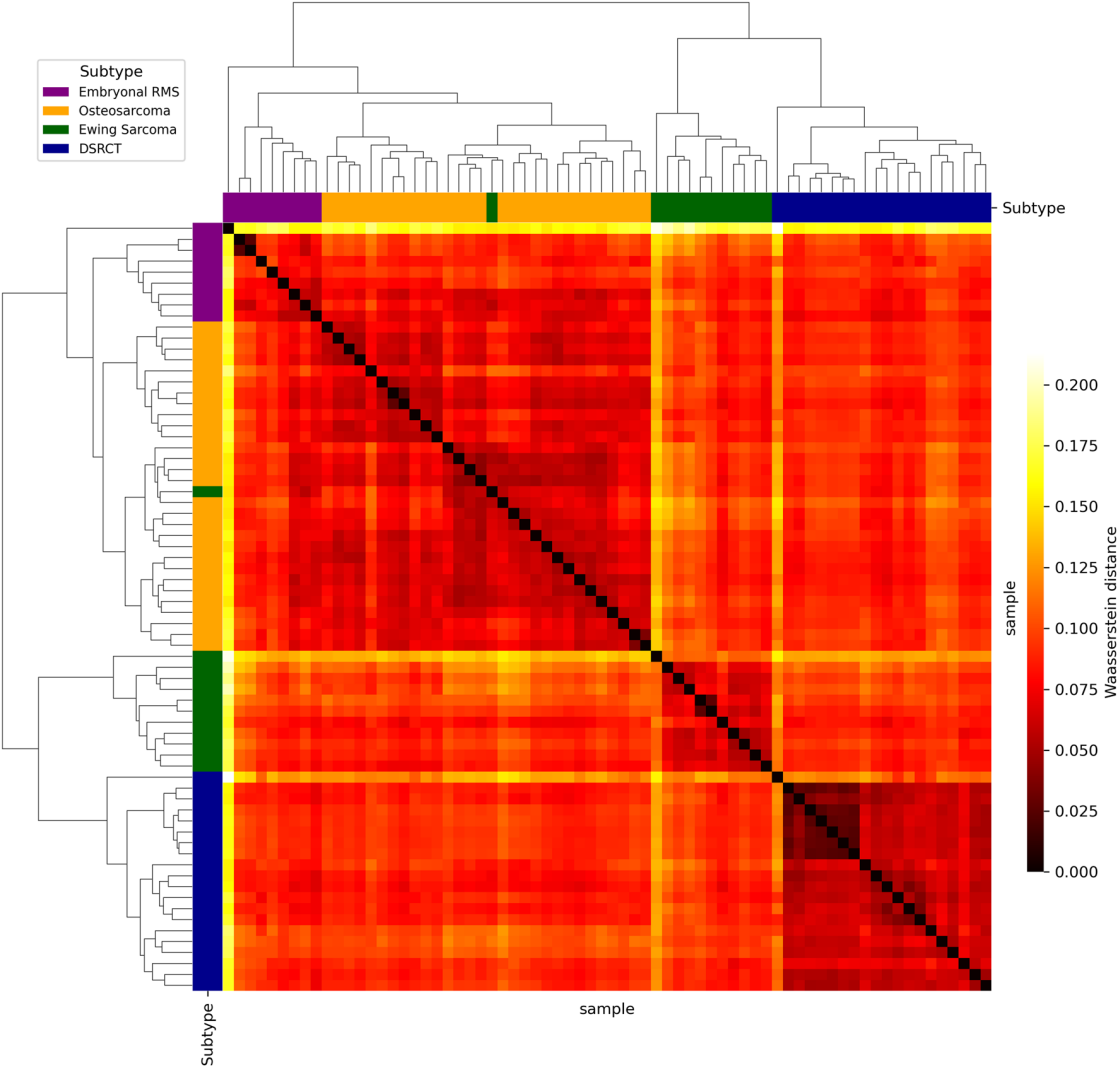
Hierarchical unsupervised OMT-Wasserstein based clustering of samples in four PS subtypes using network properties on the whole HPRD-derived graph ${\mathscr {G}}_C$. The heat map depicts the symmetric pairwise Wasserstein distance between samples. The true subtype classifications are indicated by color bars affixed to the rows and columns.

Wasserstein distance-based unsupervised hierarchical clustering was applied to cluster 70 samples from four PS subtypes using the whole HPRD-derived graph ${\mathscr {G}}_C$, described in “Graph topology” section. The resulting clustering was highly consistent with the histological subtypes and is shown in Fig. 2 with the heatmap of the pairwise Wasserstein distances. Discarding the single embryonal RMS sample outlier which did not cluster with any subtype, we used the prior knowledge that there were four molecular subtypes as a constraint on the number of clusters. The remaining 69 samples were separated into four clusters with only one misclassified sample for the histological subtypes, yielding a classification accuracy of 0.99. Of note is the incorrectly clustered EWS sample (green). Misclassification of this sample did not occur due to misdiagnosis, as it exhibited the canonical *EWSR1* - *FLI1* fusion. We suspect its low tumor purity (0.19) is the reason that this sample did not cluster with the other EWS samples. Considering that the methodology is agnostic to the histology and clinical classification, this serves as compelling evidence that the proposed approach will be helpful to understand subtype-specific biology.

**Figure 3:**
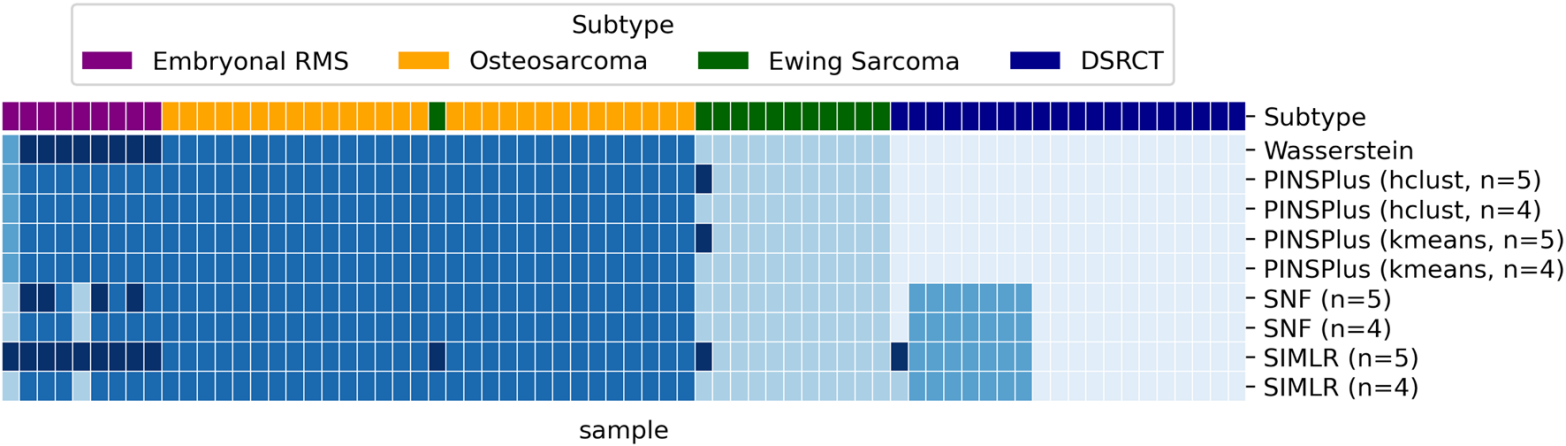
Performance of Wasserstein-based subtype clustering and benchmarked approaches. The heat map depicts the clustering partitions. Each column represents a sample and each row represents a particular subtype clustering configuration. Shades of blue indicate the clustering partition and have no associated numerical value. The true subtype classifications are indicated by the color bar above the heat map. The top row of the heat map shows the Wasserstein-based hierarchical clustering for comparison. The remaining rows are labelled by the clustering method and any pre-set parameters (e.g., internal clustering and n indicates the number of clusters). As can be seen, the Wasserstein-based clustering yields the best correspondence with the true subtypes.

We benchmarked the Wasserstein-based hierarchical clustering performance against three state-of-the-art subtyping methods: First, PINSPlus (Perturbation clustering for data INtegration and disease Subtyping) uses perturbations to combat noise and find resilient clusters when determining the optimal clustering^32^. We tested PINSPlus with both of its built-in clustering options: hierarchical clustering (hclust) and k-means (kmeans). Second, SNF (Similarity Network Fusion) was developed particularly to handle multi-omic data by fusing the different data channels^33^. Since we only have single-omic data (namely, RNA-Seq), we applied the method without the fusing step. Third, SIMLR (Single-cell Interpretation via Multi-kernel LeaRning) constructs an integrated similarity matrix by combining multiple Gaussian kernels to capture multiple representations of the data for downstream clustering^34^. Although originally presented for single-cell data analysis, the authors note that SIMLR is applicable to broader applications. Each benchmarked approach includes a heuristic for agnostically determining the optimal number of clusters. We tried each of the heuristics and found that they typically performed worse than a pre-set value of 4 or 5 when comparing the resulting clustering to the true subtypes, further justifying our choice for this pre-selection. More specifically, we found that SIMLR required too much memory for the heuristic to run and PINSPlus, depending on the configuration, only identified 1 or 2 clusters and an outlier. SNF includes 2 heuristics: (1) the eigen gap heuristic which only identified 2 clusters and (2) the rotation gap heuristic, which did a bit better and identified 5 clusters, but they did not match the true subtypes as well as the Wasserstein-based clustering. We, therefore, applied each validation method to the RNA-Seq expression profiles with the number of clusters pre-set to 4 and 5 (5 was used to allow for 1 cluster corresponding to the outlier), analogous to how we performed the Wasserstein-based clustering. The resulting clustering for each approach is shown in Fig. 3.

### Functional analysis

#### Critical curvature filter: results

**Figure 4:**
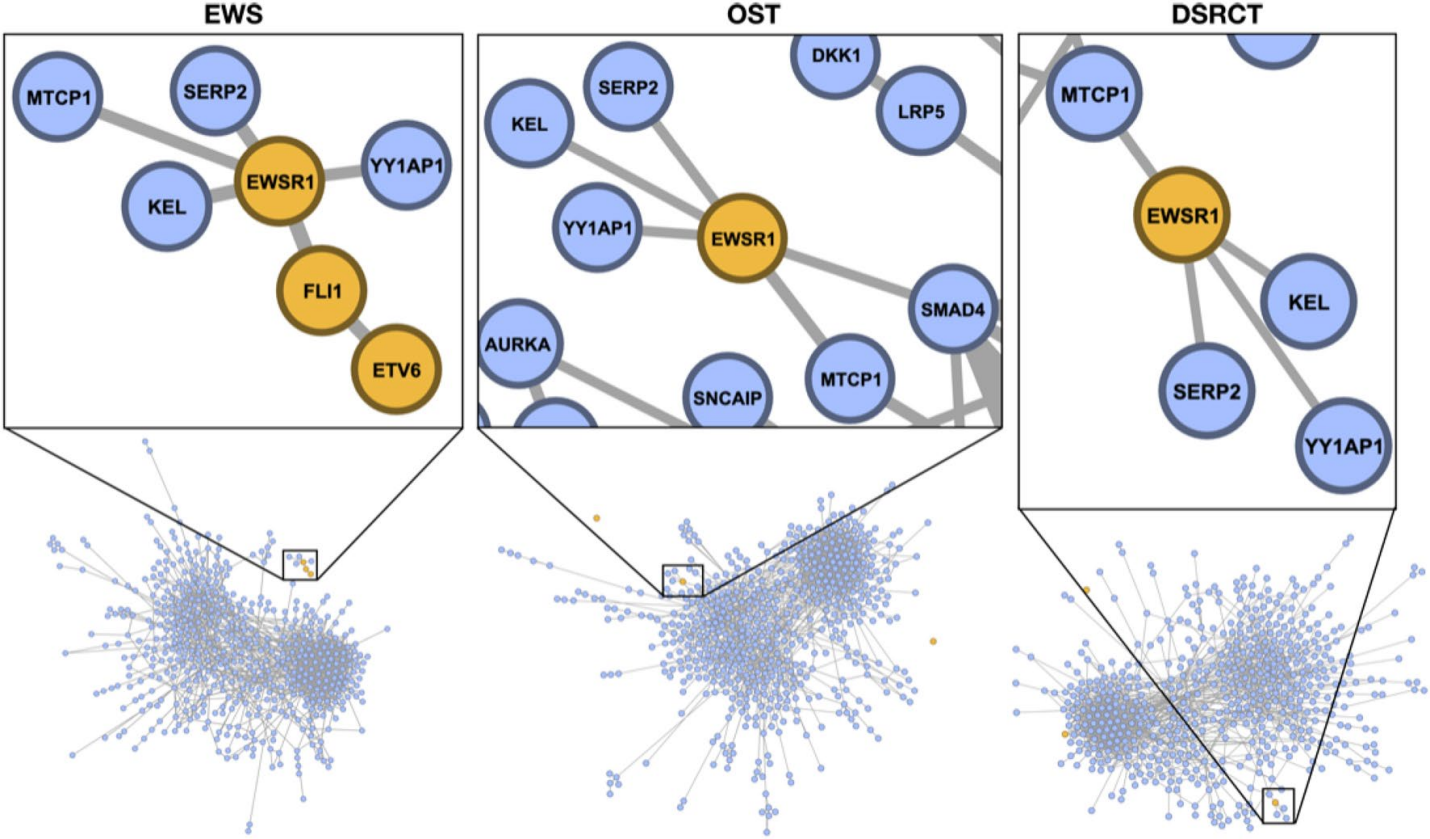
Critical curvature filtering of pediatric sarcoma networks. Functional community structures at the critical scale were realized by pruning bridges with negative critical curvature. The EWS network recovered the known functional *EWSR1*-*FLI1*-*ETV6* association.

In the EWS network, preferential community formation by filtering edges with negative critical curvature (at the determined critical mixing scale) captured the characteristic *EWSR1*-*FLI1* fusion and the novel *FLI1*-*ETV6* interaction. Finding this persistent *EWSR1*-*FLI1*-*ETV6* relationship was purely a mathematical discovery with great biological significance. This was distinctly different from the connectivity between these genes found in the OST and DSRCT networks, highlighted in Fig. 4. Removal of edges with negative critical curvature resulted in 1,481 (55.53%) remaining edges in the EWS network, 1,483 (55.61%) edges in the OST network, and 1,479 (55.46%) edges in the DSRCT network. By incrementally filtering edges by critical curvature value, we found that *EWSR1*, *FLI1* and *ETV6* form a single connected component that persists until only 587 (22.01%) edges in the EWS network remain before *ETV6* breaks away. The *EWSR1*-*FLI1* association persists further until 296 (11.10%) edges remain and a majority of the network has been decomposed.

To validate this finding implicating *ETV6* in EWS is not biased to metastatic and relapse patients, a dataset of an independent cohort consisting of 22 EWS tumors from event-free patients in the GEO database (Series GSE63157) was downloaded and analyzed. Individual GEO accession numbers can be found in the supplementary material (Table S1). The expression profiling for this dataset was performed by Affymetrix microarray. Although differences in the profiling platform and natural heterogeneity across tumors can be expected to influence the weighted network and analysis, strong biological signals should persist. The critical curvature filter analysis replicated the *FLI1*-*ETV6* associated interaction. *EWSR1* is not directly associated with *FLI1*, but remains connected via *ETV6*, where the shorted path connecting *EWSR1* to *FLI1* is: *EWSR1* $\rightarrow$ *BTK* $\rightarrow$ *CBL* $\rightarrow$ *CRKL* $\rightarrow$ *ETV6* $\rightarrow$ *FLI1*. When the first version of this paper was written, to the best of our knowledge, there was no mention implicating *ETV6* in EWS in the literature. However, a recent study independently identified *ETV6* as having a role in EWS^35^, further validating this finding.

#### Multi-scale functional clustering: results

**Figure 5:**
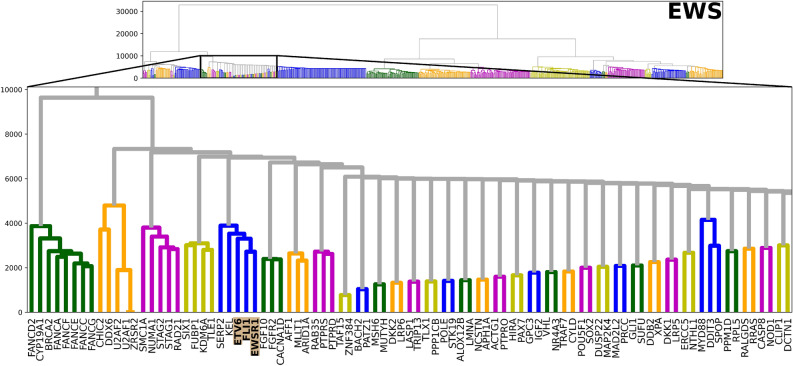
Hierarchical-acc clustering of the EWS network, highlighting the *EWSR1*-*FLI1*-*ETV6* association.

Hierarchical-acc clustering was performed on the EWS network (${\mathscr {G}}_F$). The resulting dendrogram encapsulated preferential gene clustering according to their *geometric cooperation*. As one would expect in EWS, *EWSR1*, *FLI1* and *ETV6* clustered together, as highlighted in Fig. 5. Importantly, this cluster was recovered in a purely agnostic fashion that is unique to the EWS network that would not have been found by standard approaches such as differential gene expression analysis or correlation analysis.

There are two main questions that need to be addressed when validating the methodology and this finding: (1) is the *EWSR1-FLI1-ETV6* association a EWS-specific finding or does the methodology always find this result? and (2) can the methodology identify known associations in other subtypes? To answer both questions, we applied the multi-scale Hierarchical-acc clustering to the DSRCT network. To assess the methodology’s performance, we looked at which genes were found to cluster near *EWSR1*. The resulting annotated dendrograms for EWS and DSRCT are shown in Figs. S3 and  S4, respectively. As expected, we found *FLI1* and *ETV6* cluster near *EWSR1* in the EWS network, and *WT1* clusters near *EWSR1* in the DSRCT network, with no particular converse association. We note that *WT1* does not cluster as closely to *EWSR1* in DSRCT as *FLI1* does in EWS. This may be due to the different type of interaction that *WT1* has with *EWSR1* compared to *FLI1*, where *EWSR1*-*FLI1* associate in the wild type and fused form, whereas *EWSR1* does not interact with *WT1* in the absence of the fusion. Nevertheless, *EWSR1*, *FLI1*, *ETV6*, and *WT1* show distinct and preferential functional cooperation in EWS and DSRCT. This suggests the methodology is indeed capable of finding subtype-specific biologically relevant associations, as desired, and further supports the EWS-specific role of *ETV6*.

As a side note, *KEL* and *SERP2* are leaf nodes attached to *EWSR1* in the original EWS graph, so it is not surprising that they are found in the same cluster. However, even this dependency is found to be less functionally relevant than the *EWSR1*-*FLI*-*ETV6* association, as demonstrated by the hierarchical ordering. Since the *EWSR1*-*FLI1* fusion has proven difficult to directly target^36^, we investigated how they are affected by other interactions in the network, described in the next section.

#### Edge perturbation simulations: results

Perturbation simulations were performed on each edge in the EWS network and curvature was computed for gene pairs *EWSR1*-*FLI1*, *FLI1*-*ETV6* and *EWSR1*-*ETV6* as described in “Edge perturbation simulations” section to assess the functional effect of targeted disruption in direct and indirect cooperation on the system.

The *net* change in curvature $\Delta$ between two nodes $r,s\in {\mathscr {V}}$ in response to perturbing edge $(i,j)\in {\mathscr {E}}$ measures the net change in robustness, which is quantified as the difference in curvature after (i.e., with perturbed edge weight ${\hat{w}}_{ij} = \epsilon$) and before (i.e., baseline) effectively removing communication along the perturbed edge. The sign of $\Delta$ allows us to distinguish between *strengthening* and *weakening* effects, characterized respectively by an increase or decrease in curvature with respect to the baseline. Edges that disconnect the network when removed were eliminated from consideration because the distance between nodes linked by that edge approaches infinity as the edge is perturbed, essentially breaking the communication altogether. We then ranked the effects perturbing the remaining edges had on the *EWSR1*-*FLI1*, *FLI1*-*ETV6* and *EWSR1*-*ETV6* interactions. Perturbed edges with absolute value of effect greater than $1\times 10^{-5}$ (i.e., $|\Delta | > 1\times 10^{-5}$) on the *EWSR1*-*FLI1*, *FLI1*-*ETV6* and *EWSR1*-*ETV6* interactions are listed respectively in Tables  1,  2 and  3 along with the net effect $\Delta$. This cutoff was selected due to the large drop-off of negligible effects observed smaller than $1\times 10^{-5}$. An overview of the net effects of each perturbed edge listed in these tables is shown in Fig. S5.

Additionally, line-plots of the pairwise curvatures of *EWSR1-FLI1*, *ETV6-FLI1*, and *EWSR1-ETV6* as functions of the decreasing perturbed edge weights are plotted for perturbed edges ranked in the top two largest positive and negative effects on each of the interactions (Fig. S6). The curvature for three additional perturbed edge-weights near 0 are shown to highlight trends as the edge is virtually cut. The figure references corresponding to the perturbed edges are provided in Tables 1, 2 and 3.

**Table 1:** Perturbed edges with the largest net effect ($\Delta$) on *EWSR1*-*FLI1*.

Effect	Perturbed edge	$\Delta$	Figure
Positive	*ERG*-*FLI1*	0.13028	S6a
	*EWSR1*-*ERCC5*	0.02809	S6b
	*EWSR1*-*PCBP1*	0.02442	S6c
Negative	*JUN*-*ERG*	$-$0.09351	S6d
	*AR*-*POU5F1*	$-$0.04674	S6e
	*JUN*-*AR*	$-$0.02469	
	*SMAD4*-*EWSR1*	$-$0.02456	
	*EWSR1*-*TAF1*	$-$0.02209	
	*BTK*-*EWSR1*	$-$0.02155	
	*EWSR1*-*POU5F1*	$-$0.02109	
	*EWSR1*-*FLI1*	$-$0.01803	
	*EWSR1*-*HMGA1*	$-$0.01766	
	*JUN*-*HMGA1*	$-$0.01726	
	*ETV6*-*FLI1*	$-$0.01086	S6f
	*JUN*-*TAF1*	$-$0.01042	
	*BARD1*-*EWSR1*	$-$0.00893	
	*AKT1*-*MTCP1*	$-$0.00746	
	*CRKL*-*ETV6*	$-$0.00574	
	*ETV6*-*GAB2*	$-$0.00570	S6g
	*EWSR1*-*MTCP1*	$-$0.00452	
	*AKT1*-*GAB2*	$-$0.00447	
	*JUN*-*SMAD4*	$-$0.00392	
	*CRKL*-*WAS*	$-$0.00201	
	*BTK*-*WAS*	$-$0.00201	
	*ERG*-*SETDB1*	$-$0.00168	
	*BARD1*-*SETDB1*	$-$0.00168	
	*CREBBP*-*EWSR1*	$-$0.00144	
	*HSP90AA1*-*NDRG1*	$-$0.00121	
	*MAPK1*-*HSP90AA1*	$-$0.00115	
	*MAPK1*-*GAB2*	$-$0.00115	
	*EWSR1*-*NDRG1*	$-$0.00032	
	*CREBBP*-*STAT1*	$-$0.00024	
	*JUN*-*STAT1*	$-$0.00024	
	*JUN*-*NCOA3*	$-$0.00001	
	*NCOA3*-*DDX5*	$-$0.00001	
	*DDX5*-*NDRG1*	$-$0.00001	

Abbreviations: $\Delta$: net effect or net difference in curvature (curvature with perturbed edge weight $\epsilon$ - baseline curvature).

**Table 2:** Perturbed edges with the largest net effect ($\Delta$) on *FLI1*-*ETV6*.

Effect	Perturbed edge	$\Delta$	Figure
Positive	*ERG*-*FLI1*	0.72147	S6a
	*ETV6*-*FLI1*	0.70863	S6f
	*CRKL*-*ETV6*	0.12042	
	*ETV6*-*GAB2*	0.08775	S6g
Negative	*JUN*-*ERG*	$-$0.16018	S6d
	*STAT1*-*SYK*	$-$0.03720	S6h
	*JUN*-*STAT1*	$-$0.03720	
	*SYK*-*GAB2*	$-$0.03720	
	*SOS1*-*CRK*L	$-$0.02990	
	*SOS1*-*ESR1*	$-$0.02990	
	*JUN*-*ESR1*	$-$0.02990	
	*EWSR1*-*FLI1*	$-$0.00415	
	*BTK*-*EWSR1*	$-$0.00164	
	*CRKL*-*WAS*	$-$0.00119	
	*BTK*-*WAS*	$-$0.00119	

Abbreviations: $\Delta$: net effect or net difference in curvature (curvature with perturbed edge weight $\epsilon$ - baseline curvature).

**Table 3:** Perturbed edges with the largest net effect ($\Delta$) on *EWSR1*-*ETV6*.

Effect	Perturbed edge	$\Delta$	Figure
Positive	*EWSR1*-*ERCC5*	0.02297	S6b
	*EWSR1*-*PCBP1*	0.02144	S6c
	*CRKL*-*WAS*	0.00859	
	*BTK*-*WAS*	0.00859	
	*EWSR1*-*POU5F1*	0.00383	
Negative	*ERG*-*FLI1*	$-$0.07069	S6a
	*ETV6*-*GAB2*	$-$0.05692	S6g
	*BTK*-*EWSR1*	$-$0.05339	
	*SMAD4*-*EWSR1*	$-$0.03921	
	*JUN*-*ERG*	$-$0.03168	S6d
	*AKT1*-*GAB2*	$-$0.02363	
	*JUN*-*HMGA1*	$-$0.02218	
	*AR*-*POU5F1*	$-$0.02191	S6e
	*MAPK1*-*GAB2*	$-$0.02154	
	*RB1*-*TAF1*	$-$0.01824	
	*EWSR1*-*TAF1*	$-$0.01785	
	*EWSR1*-*HMGA1*	$-$0.01492	
	*AKT1*-*AR*	$-$0.01082	
	*RB1*-*MAPK1*	$-$0.01038	
	*AKT1*-*MTCP1*	$-$0.01030	
	*CRKL*-*ETV6*	$-$0.01001	
	*EWSR1*-*MTCP1*	$-$0.00938	
	*MAPK1*-*SMAD4*	$-$0.00874	
	*BARD1*-*EWSR1*	$-$0.00448	
	*ETV6*-*FLI1*	$-$0.00414	S6f
	*EWSR1*-*FLI1*	$-$0.00394	
	*BRCA1*-*BARD1*	$-$0.00275	
	*HSP90AA1*-*NDRG1*	$-$0.00230	
	*EWSR1*-*NDRG1*	$-$0.00223	
	*MAPK1*-*HSP90AA1*	$-$0.00139	
	*CREBBP*-*EWSR1*	$-$0.00125	
	*BRCA1*-*AKT1*	$-$0.00121	
	*ERG*-*SETDB1*	$-$0.00021	
	*BARD1*-*SETDB1*	$-$0.00021	
	*CREBBP*-*NCOA1*	$-$0.00010	
	*MAPK1*-*NCOA1*	$-$0.00010	

Abbreviations: $\Delta$: net effect or net difference in curvature (curvature with perturbed edge weight $\epsilon$ - baseline curvature).

#### Gene knockout simulations: results

To assess the functional effect of targeted gene disruption on the system, gene knockout simulations were performed on each gene in the EWS network (excluding *EWSR1*, *FLI1* and *ETV6*) and curvature was computed for gene pairs *EWSR1*-*FLI1*, *FLI1*-*ETV6* and *EWSR1*-*ETV6* as described in “Gene knockout simulations” section. In a similar manner to the edge perturbation simulations, the net change in curvature $\Delta$ between two nodes before and after removing a node from the network measures the net change in robustness resulting from the simulated gene knockout. We ranked the effect knocking out each gene had on the *EWSR1*-*FLI1*, *FLI1*-*ETV6* and *EWSR1*-*ETV6* interactions. Not surprisingly, simulated knockout of genes outside of a 2-hop radius of *EWSR1*, *FLI1* and *ETV6* had negligible effects on their interactions by virtue of the way the nodal measures are constructed. Also not surprisingly, simulated knockout of all genes neighboring (i.e., within a 1-hop radius) *EWSR1*, *FLI1* and *ETV6* had non-negligible effects on their interactions. However, a non-immediately obvious result was that simulated knockout of only some of the genes in a 2-hop radius of *EWSR1*, *FLI1* and *ETV6* affected their interactions. These genes may serve as potential candidates for therapeutic intervention.

**Figure 6:**
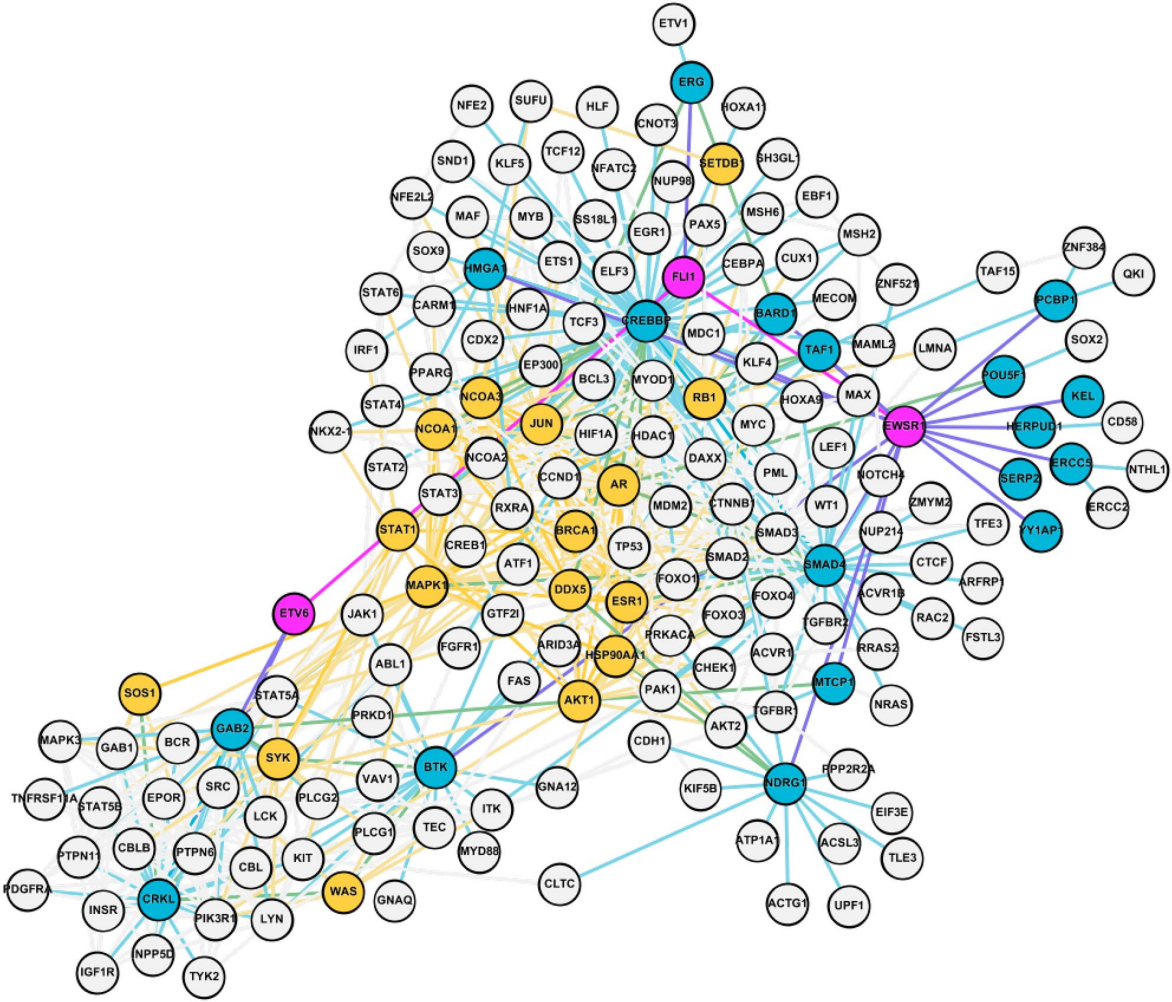
Subgraph containing *EWSR1*, *FLI1*, *ETV6* and their 2-hop neighborhoods. *EWSR1*, *FLI1* and *ETV6* are shown in magenta, candidates in their one-hop neighborhoods are shown in blue and candidates in their two-hop neighborhoods are shown in yellow. The remaining nodes in their 2-hop neighborhoods that are not candidates are shown in light gray. Edges are colored by the average of their respective incident nodes.

An overview of the potential candidate gene targets whose simulated knockout affected the *EWSR1*-*FLI1*, *FLI1*-*ETV6* and *EWSR1*-*ETV6* interactions is shown in Fig. S7 and a sub-network of *EWSR1*, *FLI1* and *ETV6* with genes within a 2-hop radius is shown in Fig. 6. The ranked effects of genes with absolute value of knockout effect greater than $1\times 10^{-5}$ (i.e., $|\Delta |>1\times 10^{-5}$) on the *EWSR1*-*FLI1*, *FLI1*-*ETV6* and *EWSR1*-*ETV6* interactions are listed in Tables 4, 5, and 6, respectively, along with the knockout effect $\Delta$.

#### Predicted candidate therapeutic target prioritization

Determining the most viable of the predicted candidate therapeutic targets is crucial for guiding cost and time-efficient experimentation. To identify the most therapeutically relevant targets, we used the DepMap portal (https://depmap.org/portal/) to prioritize predicted genes with verified actionable structures. Out of the 34 predicted candidate targets appearing in Tables 1, 2, 3, 4, 5, 6, 21 are known to have a druggable structure (*AKT1, AR, BARD1, BRCA1, BTK, CREBBP, ERCC5, ESR1, HERPUD1, HSP90AA1, JUN, MAPK1, NCOA1, POU5F1, RB1, SETDB1, SMAD4, SOS1, STAT1, SYK, TAF1*), and are therefore referred to as the priority-candidates. Of the priority candidates, 6 were found with enriched dependency in Ewing Sarcoma cell lines (*AKT1, BARD1, HSP90AA1, NCOA1, SETDB1, SMAD4*). Furthermore, several of the priority-candidates are annotated in OncoKB as targetable with an FDA approved drug (*AKT1*, *BRCA1*, *BTK*, *ESR1*).

**Table 4:** Knocked-out genes with the largest net effect ($\Delta$) on *EWSR1*-*FLI1*.

Effect	Knocked-out gene	$\Delta$
Positive	*ERG*	0.13028
	*ERCC5*	0.02809
	*PCBP1*	0.02442
	*YY1AP1*	0.02418
	*SERP2*	0.01771
	*KEL*	0.01584
	*HERPUD1*	0.01077
Negative	*JUN*	-0.09351
	*AR*	$-$0.04674
	*SMAD4*	$-$0.02456
	*TAF1*	$-$0.02209
	*BTK*	$-$0.02155
	*POU5F1*	$-$0.02109
	*HMGA1*	$-$0.01766
	*BARD1*	$-$0.00893
	*AKT1*	$-$0.00746
	*CRKL*	$-$0.00574
	*GAB2*	$-$0.00570
	*MTCP1*	$-$0.00452
	*WAS*	$-$0.00201
	*SETDB1*	$-$0.00168
	*CREBBP*	$-$0.00144
	*HSP90AA1*	$-$0.00121
	*MAPK1*	$-$0.00115
	*NDRG1*	$-$0.00032
	*STAT1*	$-$0.00024
	*NCOA3*	$-$0.00001
	*DDX5*	$-$0.00001

Abbreviations: $\Delta$: net effect or net difference in curvature (curvature after gene knockout - baseline curvature).

**Table 5:** Knocked-out genes with the largest net effect ($\Delta$) on *FLI1*-*ETV6*.

Effect	Knocked-out gene	$\Delta$
Positive	*ERG*	0.72148
	*CRKL*	0.12042
	*GAB2*	0.08775
Negative	*JUN*	$-$0.16018
	*STAT1*	$-$0.03720
	*SYK*	$-$0.03720
	*SOS1*	$-$0.02990
	*ESR1*	$-$0.02990
	*BTK*	$-$0.00164
	*WAS*	$-$0.00119

Abbreviations: $\Delta$: net effect or net difference in curvature (curvature after gene knockout - baseline curvature).

**Table 6:** Knocked-out genes with the largest net effect ($\Delta$) on *EWSR1*-*ETV6*.

Effect	Knocked-out gene	$\Delta$
Positive	*ERCC5*	0.02297
	*PCBP1*	0.02144
	*YY1AP1*	0.02133
	*SERP2*	0.01769
	*KEL*	0.01616
	*HERPUD1*	0.01153
	*WAS*	0.00859
	*POU5F1*	0.00383
Negative	*ERG*	$-$0.07069
	*GAB2*	$-$0.05692
	*BTK*	$-$0.05339
	*SMAD4*	$-$0.03921
	*JUN*	$-$0.03168
	*AKT1*	$-$0.02363
	*AR*	$-$0.02191
	*MAPK1*	$-$0.02154
	*RB1*	$-$0.01824
	*TAF1*	$-$0.01785
	*HMGA1*	$-$0.01492
	*CRKL*	$-$0.01001
	*MTCP1*	$-$0.00938
	*BARD1*	$-$0.00448
	*BRCA1*	$-$0.00275
	*HSP90AA1*	$-$0.00230
	*NDRG1*	$-$0.00223
	*CREBBP*	$-$0.00125
	*SETDB1*	$-$0.00021
	*NCOA1*	$-$0.00010

Abbreviations: $\Delta$: net effect or net difference in curvature (curvature after gene knockout - baseline curvature).

Lastly, we performed gene set enrichment analysis (using all gene sets available in MSigDB^37^) on the 34 predicted candidate targets to gain insight on what the primary cellular functions and pathways the predicted targets are involved in. Two out of the top five enriched gene sets (Table S2) involve RNA Polymerase II activity. This complements the already established interaction between the *EWSR1*-*FLI1* fusion and *EWSR1* alone with RNA Polymerase II^38^. Finding that the candidate gene targets, whose simulated perturbation affected the *EWSR1*-*FLI1*-*ETV6* association, are involved in known *EWSR1*-*FLI1* fusion interactions, suggests their ability to disrupt *EWSR1*-*FLI1* fusion behavior and gives further credence to the proposed methodology.

## Discussion

In this work, we utilized a network version of the geometric concept of curvature to model information variability, robustness, and dysregulation of cancer gene networks. PSs represent a phenotypically diverse group of malignant solid tumors^39^. A subset of PS is characterized by oncogenic driver fusion genes such as EWS-FLI1 in EWS, EWS-WT1 in DSRCT, and PAX3/7-FOXO1 in fusion-positive rhabdomyosarcoma^40^. Given the heterogeneous nature of PS and often overlapping microscopic structural features (histology) across different PS subtypes, the presence and detection of driver fusion genes in PS has aided in the diagnostic classification of these tumors. Here, we demonstrated that analysis of the curvature using RNA-Seq gene expression profiles as a function of scale is able to define robust networks that distinguish subtypes of PS. These approaches may therefore serve as a genomic-based classifier aiding the diagnosis of PS subtypes.

Given the lack of other driver mutations that typify the mutational landscape of PS, or pediatric tumors in general, direct targeting of fusion oncogenes has seemed a logical strategy for treating fusion-positive PS^36^. However, development of drugs that can selectively target and inhibit the activity of fusion oncogenes has remained elusive^36^. Therefore, development of strategies that identify targets that indirectly disrupt the key functional interactions nucleated by “undruggable” fusion oncoproteins, or enable the identification of driver mutations amidst a low tumor mutational landscape characteristic of pediatric cancers^41^, addresses a critical unmet need in pediatric oncology.

The work presented provides a novel approach for mining genomic sequencing data to aid diagnostic classification of PS and identify potential therapeutic targets not readily accessible by merely cataloguing a tumor’s set of mutations. This study has three main limitations. The first limitation is validation, a common challenge for computational approaches. Systematic selective targeting of genes involved in critical interactions (e.g., *EWSR1*-*FLI1*-*ETV6* interaction) and the functional consequences of inhibiting critical interactions in *in vivo* tumor models of PS will provide future validation of this approach and inform future applications of curvature analysis in pediatric oncology. The second limitation is that this study is based on RNA-Seq data, due to data availability, so we cannot measure the expression level of the fused EWS/FLI1 gene or established protein-level activity^38^. We should note that fusion oncogenes function differently than the wild type genes that comprise the fusion, exhibiting different transcriptional programs^42^. The third limitation is that this study uses the HPRD to construct the network topology of the wild type constituents of the fusion oncogenes. While curvature analysis quantifies changes in geometry of the biological networks with a fixed topology, future work is needed to account for possible topological changes.

Notwithstanding these limitations, the methodology demonstrated promising capability to detect and inform on subtype-specific relevant functional associations among genes. Moreover, the nature of the limitations restricts the analysis to genes and HPRD documented interactions. Therefore, other known interactions with *EWSR1* and the *EWSR1*-*FLI1* fusion, e.g., RNA Polymerase II, are not explicitly represented in the data or methodology. Yet, gene set enrichment analysis identifies RNA Polymerase II activity in EWS, suggesting that the network-level behavior can inform on implicit biological behavior using incomplete and non-specialized interactions.

The dynamic network curvature analytical framework formulated in the present work is well-suited for analyzing data sets with a small number of samples, as is common in clinical studies. Due to its parameter-free nature, the proposed methodology is not susceptible to over-fitting, which contributes to its appeal. Additionally, the framework is clearly applicable to any number of network problems of interest in cancer research. In particular, we plan to explore the network changes leading from ductal carcinoma in situ (DCIS) breast cancer to invasive ductal carcinoma (IDC) in future work.

### Supplementary Information

Supplementary Information.

## Data Availability

The RNA-Seq data generated in this study have been deposited into the Sequence Read Archive (SRA) database under the following accession numbers: SAMN38494083 - SAMN38494152, associated with BioProject ID: PRJNA1046425.

## Code availability

The code written in Python may be found at https://github.com/MSK-MOI/dynosarc.
