# Supplementary material for: Dynamic network curvature analysis of gene expression reveals novel potential therapeutic targets in sarcoma
Source: Sci Rep. 2024 Jan 4;14:488. doi: 10.1038/s41598-023-49930-4 (PMC10766622; doi:10.1038/s41598-023-49930-4)
Supplement: Supplementary file 1 — Supplementary Information. [file 41598_2023_49930_MOESM1_ESM.pdf]

Dynamic Network Curvature Analysis of Gene Expression Reveals Novel Potential Therapeutic Targets in Sarcoma

Rena Elkin[1], Jung Hun Oh[1], Filemon Dela Cruz[2], Larry Norton[3], Joseph O. Deasy[1], Andrew L. Kung[2], and Allen R. Tannenbaum[4*]

[1]Department of Medical Physics, Memorial Sloan Kettering Cancer Center, New York, 10065, USA
[2]Department of Pediatrics, Memorial Sloan Kettering Cancer Center, New York, 10065, USA
[3]Department of Medicine, Memorial Sloan Kettering Cancer Center, New York, 10065, USA
[4]Departments of Computer Science and Applied Mathematics & Statistics, Stony Brook University, Stony Brook, 11794, USA
[*]Corresponding author: allen.tannenbaum@stonybrook.edu

Supplemental Material

GSM1542334
GSM1542335
GSM1542336
GSM1542337
GSM1542338
GSM1542341
GSM1542342
GSM1542345
GSM1542347
GSM1542348
GSM1542352
GSM1542354
GSM1542355
GSM1542357
GSM1542358
GSM1542359
GSM1542369
GSM1542372
GSM1542374
GSM1542375
GSM1542376
GSM1542379

Table S1. GEO accession numbers for 22 event-free EWS tumors (Series GSE63157).

Gene set	Description	FDR p-value
Neoplasm	An organ or organ-system abnormality that consists of uncontrolled autonomous cell-proliferation which can occur in any part of the body as a benign or malignant neoplasm (tumor).	3.17×10^{-15}
RNA Polymerase II specific DNA binding transcription factor binding	Binding to a sequence-specific DNA binding RNA polymerase II transcription factor, any of the factors that interact selectively and non-covalently with a specific DNA sequence in order to modulate transcription.	7.25×10^{-15}
IL-2 signaling pathway	IL-2 signaling pathway.	6.27×10^{-14}
Positive regulation of transcription by RNA Polymerase II	Any process that activates or increases the frequency, rate or extent of transcription from an RNA polymerase II promoter.	$6.27 \times 10^{-}14$
Positive regulation of macromolecule biosynthetic processes	Any process that increases the rate, frequency or extent of the chemical reactions and pathways resulting in the formation of a macromolecule, any molecule of high relative molecular mass, the structure of which essentially comprises the multiple repetition of units derived, actually or conceptually, from molecules of low relative molecular mass.	1.26×10^{-13}

Table S2. Gene set enrichment on predicted candidate therapeutic targets. Two of the top five enriched gene sets are associated with RNA polymerase II.

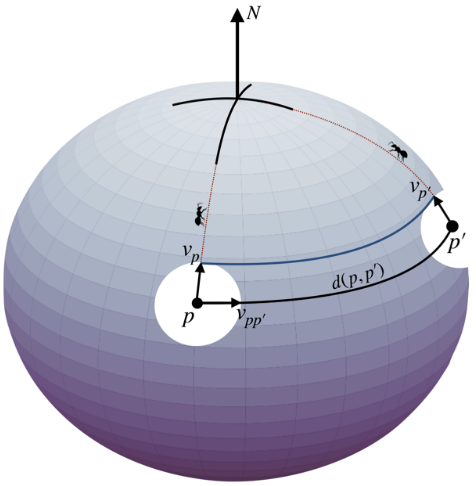

Figure S1. Geodesics obtained by parallel transport converge on a space with positive curvature. To see that the sphere has positive curvature, consider a point p, two tangent vectors v_p and $v_{pp'}$ at p perpendicular to each other and another point p' obtained by parallel transport of v_p in the direction of $v_{pp'}$ which results in the tangent vector $v_{p'}$ at p'. Then the geodesic from p in the direction of v_p will converge with the geodesic from p' in the direction $v_{p'}$ at the top of the sphere.

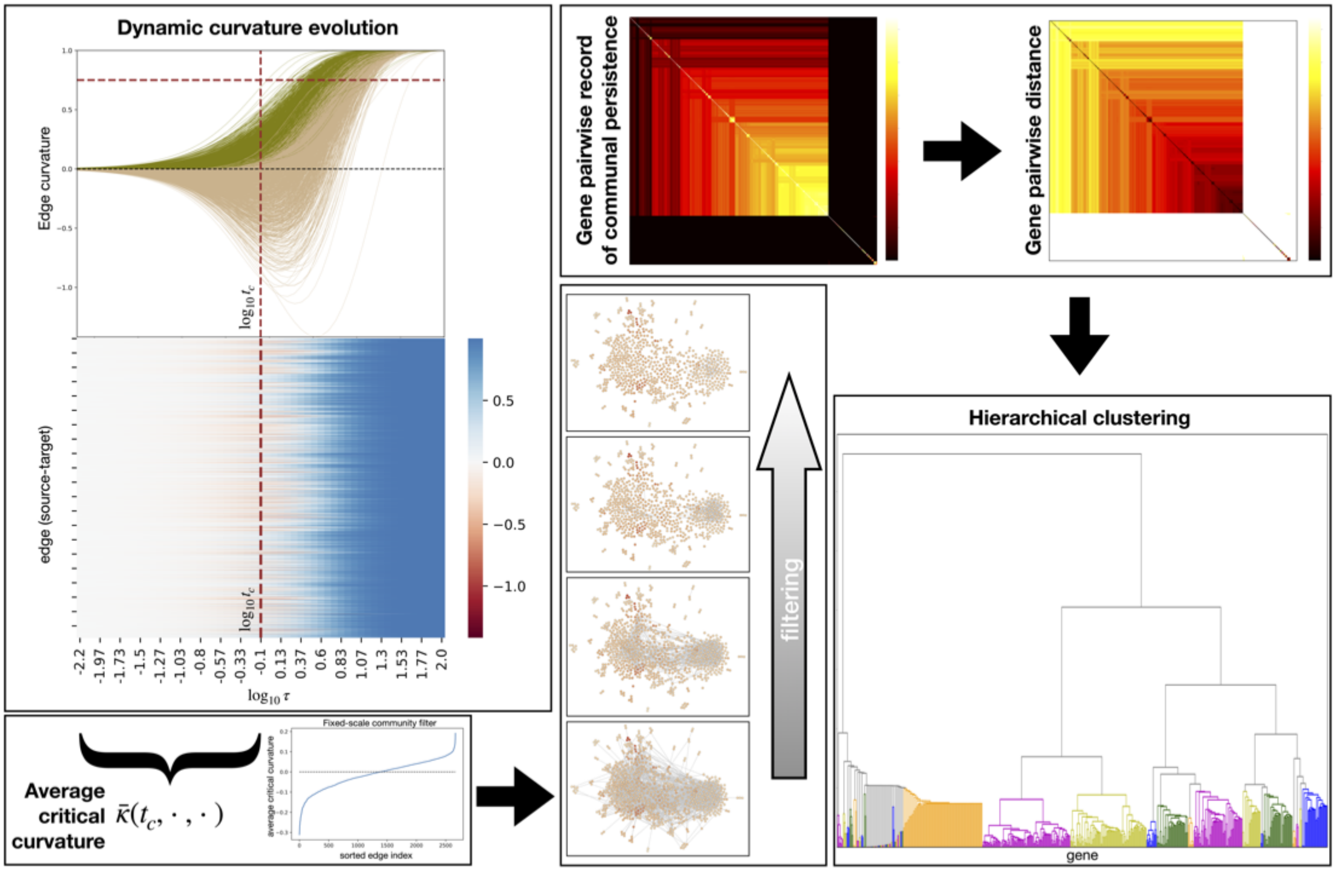

Figure S2. Hierarchical dynamic curvature clustering pipeline.

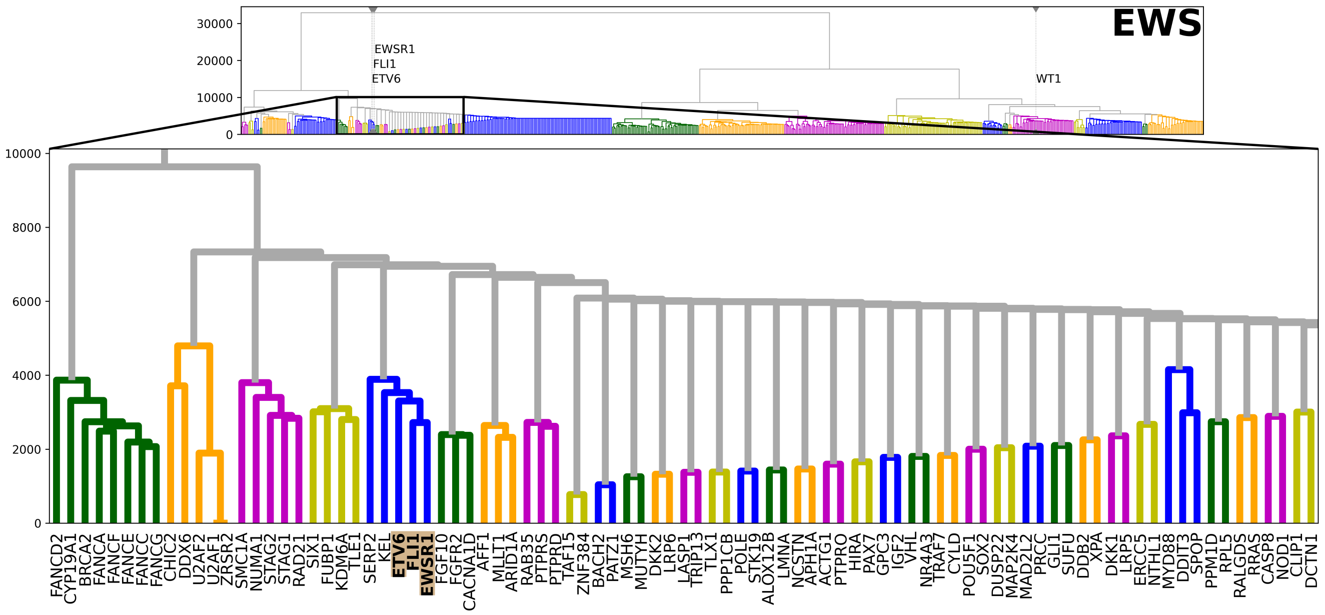

Figure S3. Hierarchical-acc clustering of the EWS network, highlighting the *EWSR1-FLI1-ETV6* association. The annotated vertical lines indicate where *EWSR1, FLI1, ETV6* and *WT1* appear in the dendrogram.

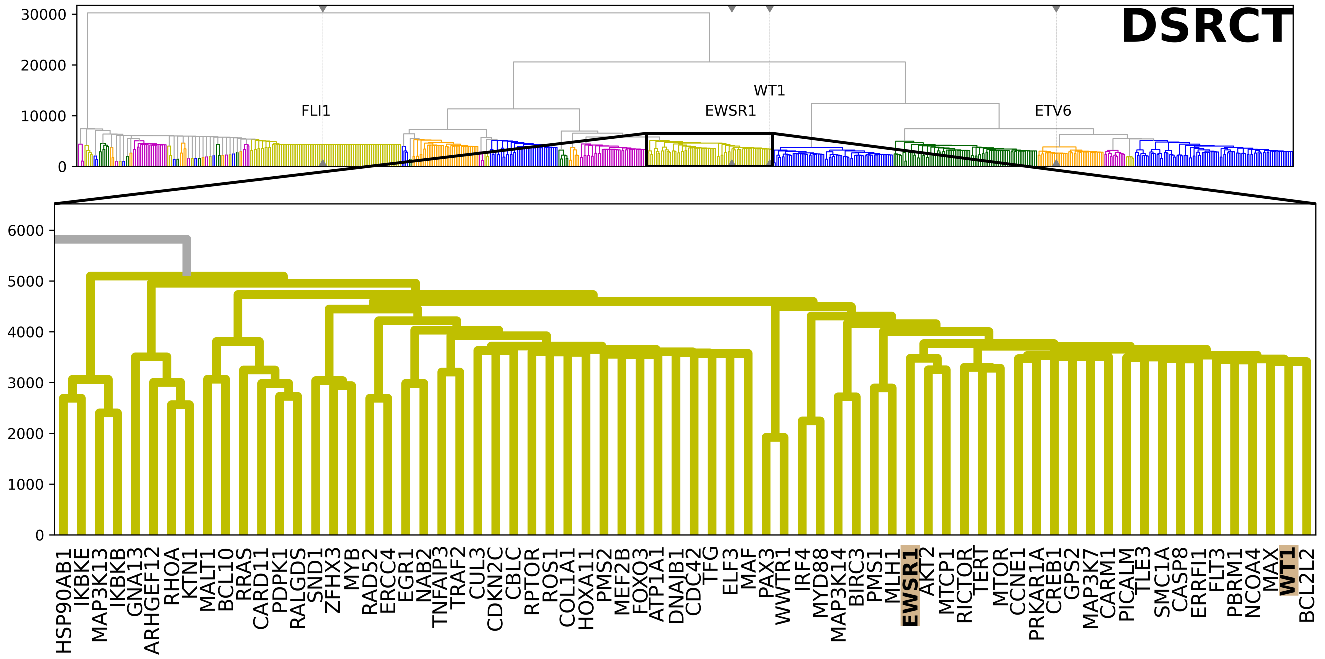

Figure S4. Hierarchical-acc clustering of the DSRCT network, highlighting the proximity between *EWSR1* and *WT1*, unique to the DSRCT network.

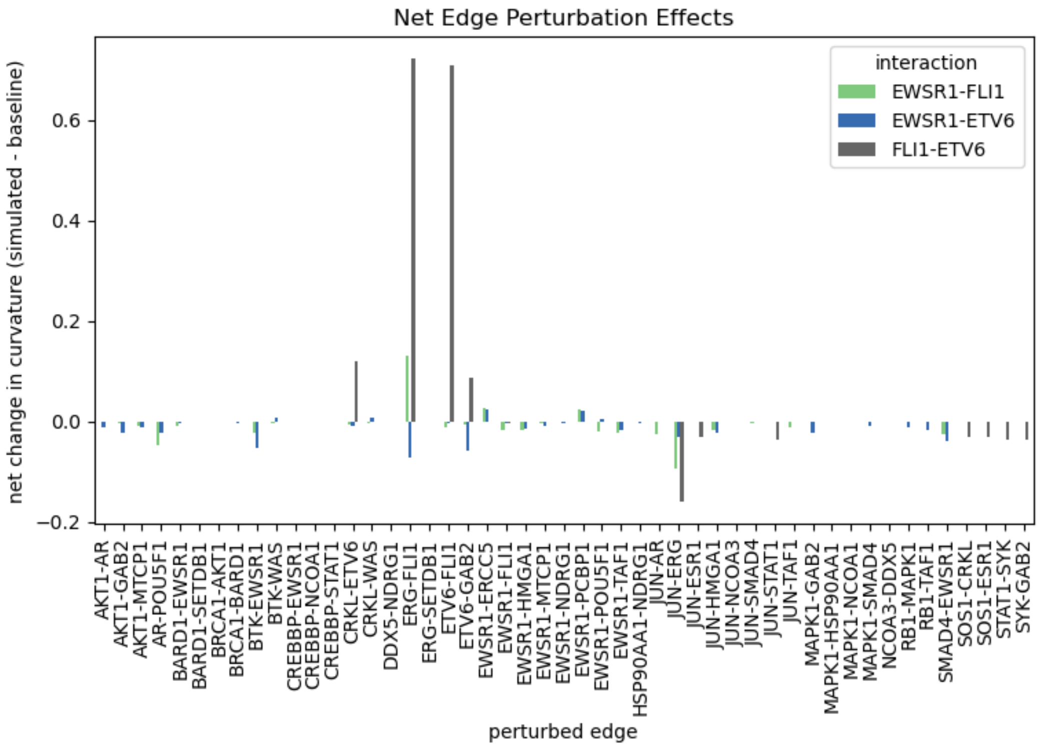

Figure S5. Net effects of simulated edge perturbations on *EWSR1-FLI1*, *EWSR1-ETV6* and *ETV6-FLI1* interactions.

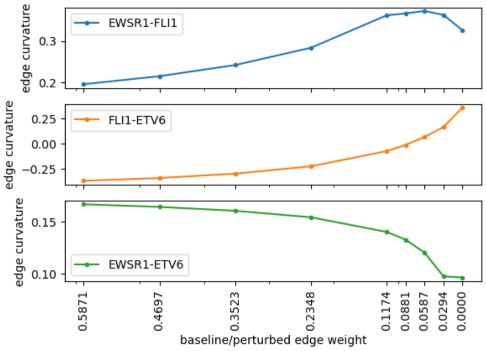

(a) Simulated perturbation of edge ERG-FLI1

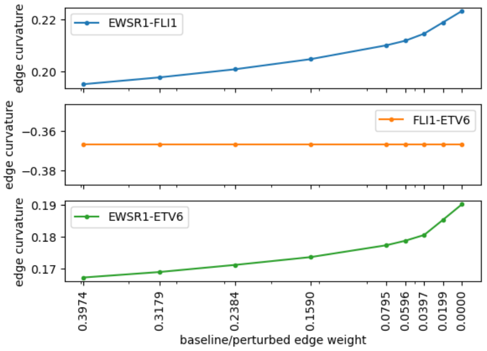

(b) Simulated perturbation of edge EWSR1-ERCC5

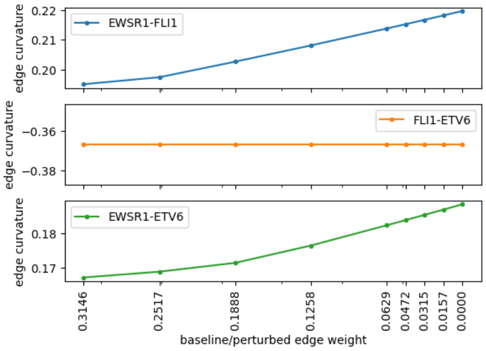

(c) Simulated perturbation of edge EWSR1-PCBP1

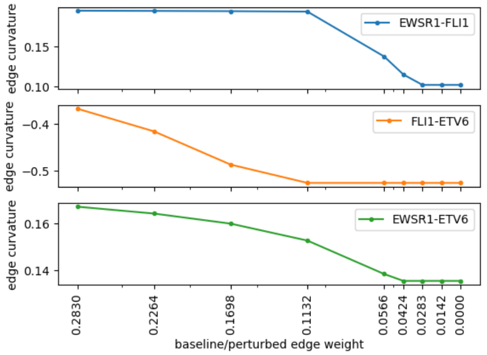

(d) Simulated perturbation of edge JUN-ERG

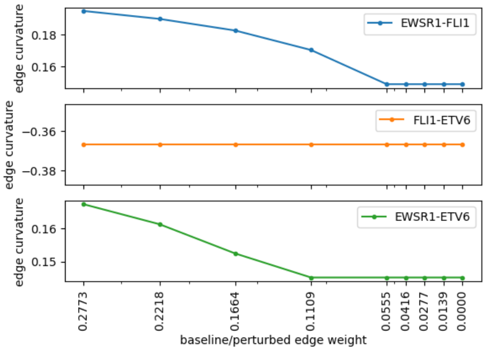

(e) Simulated perturbation of edge AR-POU5F1

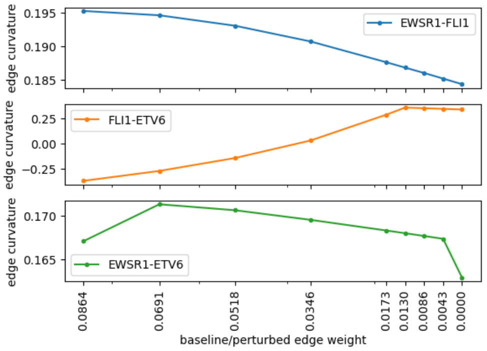

(f) Simulated perturbation of edge ETV6-FLI1

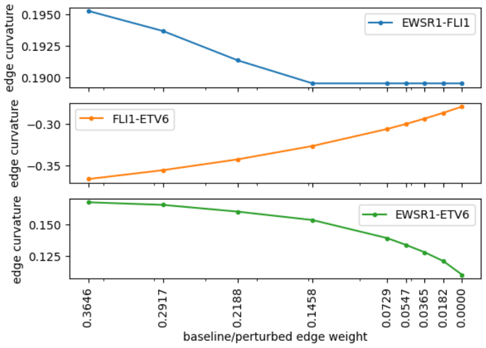

(g) Simulated perturbation of edge ETV6-GAB2

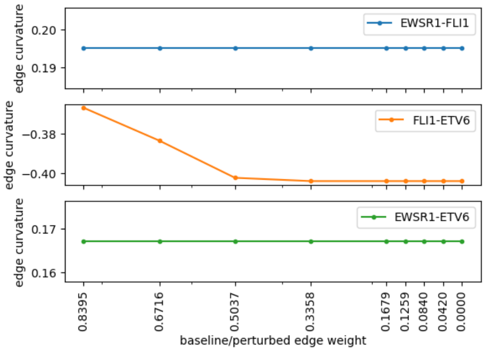

(h) Simulated perturbation of edge STAT1-SYK

Figure S6. Simulated edge perturbations.

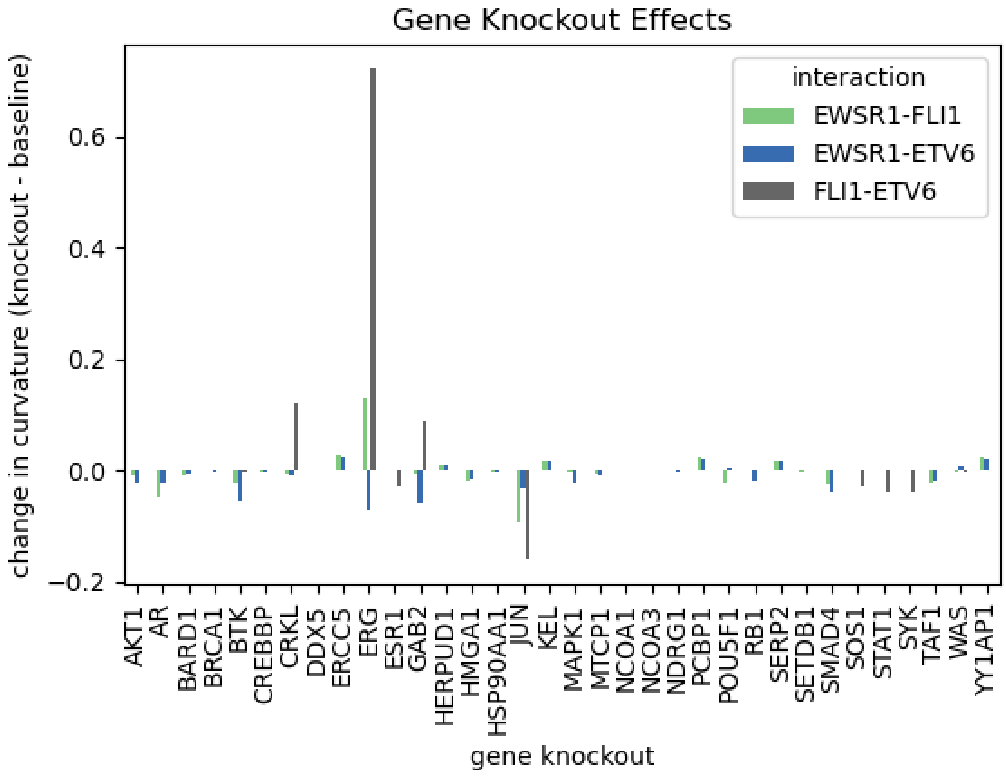

Figure S7. Effects of simulated gene knockouts on *EWSR1-FLI1*, *EWSR1-ETV6* and *ETV6-FLI1* interactions.
